# Dataset of human medial temporal lobe neurons, scalp and intracranial EEG during a verbal working memory task

**DOI:** 10.1038/s41597-020-0364-3

**Published:** 2020-01-21

**Authors:** Ece Boran, Tommaso Fedele, Adrian Steiner, Peter Hilfiker, Lennart Stieglitz, Thomas Grunwald, Johannes Sarnthein

**Affiliations:** 1 Klinik für Neurochirurgie, UniversitätsSpital und Universität Zürich, 8091 Zürich, Switzerland; 2 Neuroscience Center Zurich, ETH Zürich, Zürich, Switzerland; 3 Institute for Cognitive Neuroscience, National Research University Higher School of Economics, Moscow, Russian Federation; 4 Schweizerisches Epilepsie-Zentrum, 8008 Zürich, Switzerland; 5 Klinik für Neurologie, UniversitätsSpital Zürich, 8091 Zürich, Switzerland

**Keywords:** Electroencephalography - EEG, Magnetic resonance imaging, Cognitive neuroscience, Working memory, Hippocampus

## Abstract

We present an electrophysiological dataset recorded from nine subjects during a verbal working memory task. Subjects were epilepsy patients undergoing intracranial monitoring for the localization of epileptic seizures. Subjects performed a modified Sternberg task in which the encoding of memory items, maintenance, and recall were temporally separated. The dataset includes simultaneously recorded scalp EEG with the 10–20 system, intracranial EEG (iEEG) recorded with depth electrodes, waveforms and spike times of neurons recorded in the medial temporal lobe, and localization information on the depth electrodes. Subject characteristics and information on each trial (set size, match/mismatch, correct/incorrect, response, and response time) are given. For technical validation of data, we provide spike sorting quality metrics and the spectra of iEEG and scalp EEG signals. This dataset enables the investigation of working memory by providing simultaneous scalp EEG and iEEG recordings, which can be used for connectivity analysis, along with hard-to-obtain neuronal recordings from humans.

## Abstract (summary)

**Table:** 

Measurement(s)	brain electrical activity • Working Memory • medial temporal lobe • neuronal action potential
Technology Type(s)	electroencephalography (EEG) • microelectrode
Factor Type(s)	set size • match/mismatch • seizure onset zone
Sample Characteristic - Organism	Homo sapiens

Machine-accessible metadata file describing the reported data: 10.6084/m9.figshare.11500485

## Background & Summary

Working memory (WM) describes our capacity to prospectively store sensory input and translate it into an appropriate behavioral response. This capacity is necessary for several higher-order cognitive functions, such as reading and dialing a telephone number^1^. The anatomical basis of WM involves a widespread network of brain areas as shown noninvasively with EEG^2–6^, functional magnetic resonance imaging (fMRI)^7^ and invasively with intracranial EEG (iEEG)^8–13^. The hippocampus has been proposed to be a subcortical node associated with frontal theta oscillations^14^. Evidence for hippocampal involvement in WM originated from iEEG studies that revealed changes in theta and gamma power^10^ and cross-frequency coupling that was load-dependent during periods of WM activation^15^. The basic process that underlies the temporary storage of information in WM is persistent neuronal firing in a widespread neural network. Sustained neuronal firing - while information is maintained during the delay before a response - is a hallmark for WM^13,16–19^.

**Fig. 1:**
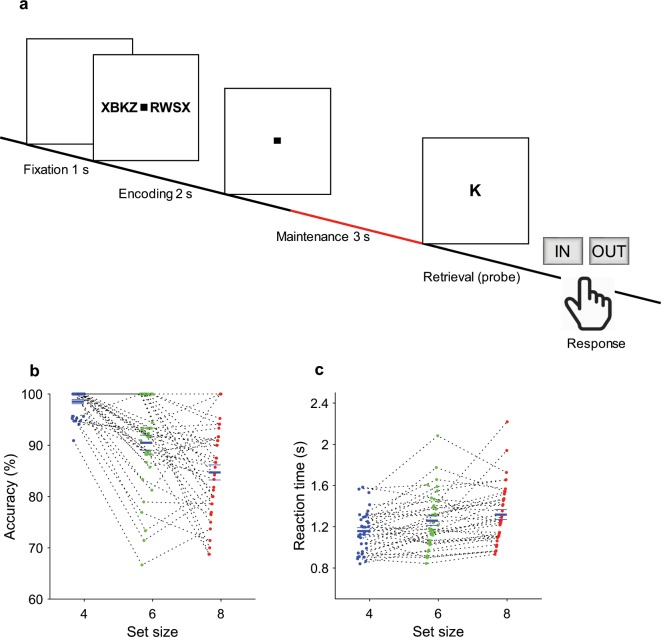
Task. **(a)** In each trial, a set of consonants was presented and had to be memorized. The set size (4, 6 or 8 letters) determined WM workload. The stimulus presentation (encoding period, 2 s) was followed by a delay (maintenance period, 3 s). After the delay, a probe letter was presented, and subjects indicated whether the probe letter was contained in the stimulus.

**Fig. 2:**
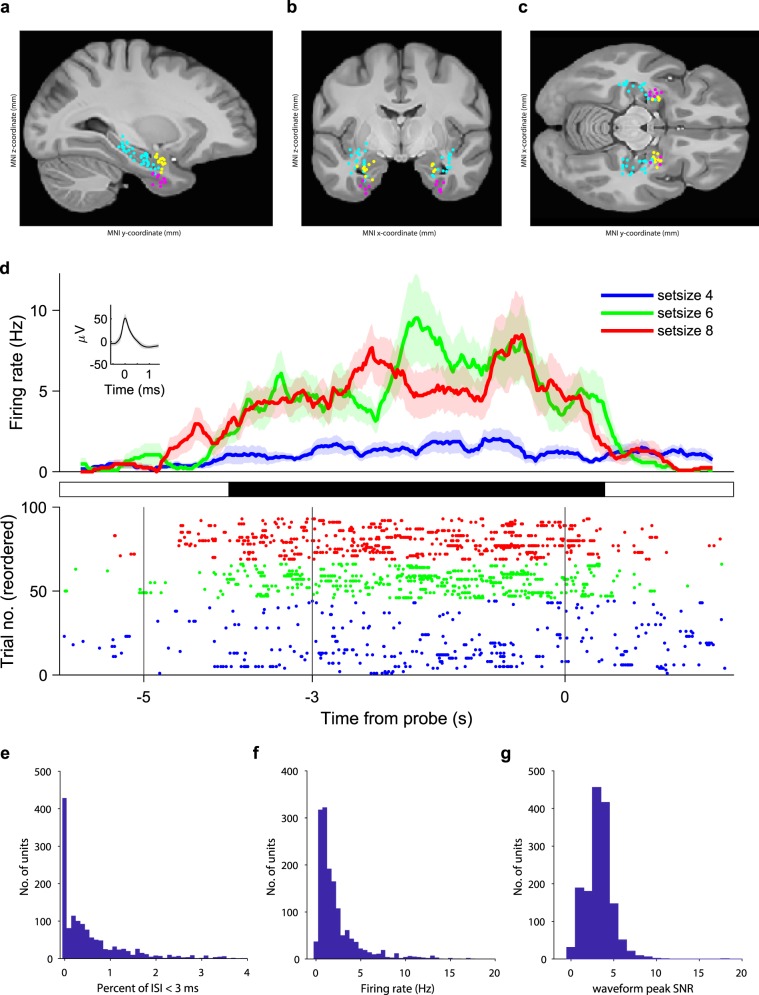
Recording sites, neuronal firing and spike sorting quality metrics. Location of the microelectrodes at the tip of the depth electrodes in Montreal Neurological Institute’s MNI152 space projected on the **(a)** parasagittal plane (x = −25.2 mm), **(b)**, coronal plane (y = −7.0 mm), **(c)** transverse plane (z = −12.4 mm) and color coded (cyan, hippocampus; magenta, entorhinal cortex; yellow, amygdala). **(d)** Example neuron. Top: (Peristimulus time histogram, bin size, 500 ms, step size, 20 ms). Shaded areas represent ± s.e.m. across trials of all spikes associated with the neuron. Inset: mean extracellular waveform ± s.e.m. Middle: periods of significance (black) between low-workload trials (set size 4, 45 trials) and high-workload trials (set sizes 6 and 8, 46 trials, P < 0.05, cluster based non-parametric permutation test). Bottom: raster plot of trials reordered to set size and RT for plotting purposes only. Compared to set size 4 (blue), the neuron fires more for set size 6 (green) and set size 8 (red). **(e)** Histogram of percentage of inter-spike intervals (ISI) < 3 ms. The majority of neurons had less than 0.5% of short ISI. **(f)** Histogram of average firing rate for all neurons. **(g)** Histogram of the signal-to-noise ratio (SNR) of the peak of the mean waveform.

We used a WM task (Fig. 1a) known to elicit frontal theta and parietal alpha oscillations during the maintenance period^2,6,13^ to clarify both the involvement of the hippocampus in the WM network and the mechanism of the functional hippocampal-cortical interaction. We recorded single- and multiunit activity from microelectrodes and iEEG in the medial temporal lobe (MTL, which includes the hippocampus, entorhinal cortex and amygdala) simultaneously with scalp EEG during the presurgical evaluations in patients with epilepsy (Fig. 2a–c).

Here we provide the data collected during the task. We have used this data to show direct evidence for hippocampal involvement in working memory^13^. The dataset has extensive reuse value, as it combines neuronal firing activity in the MTL with simultaneously recorded iEEG and scalp EEG, all in a verbal task that is feasible only for human subjects. The presence of microelectrodes allows investigating firing activity with precise timing. The wide coverage of recording sites allows investigating long-range communication between regions in cortical networks.

## Methods

### Task

We used a modified Sternberg task in which the encoding of memory items, maintenance and recall are temporally separated (Fig. 1a)^2,6^. The task has been described in detail earlier^2,13^. Each stimulus set consisted of eight consonants at the center of the screen, presented for 2 seconds. The middle four, six or eight letters were memory items, corresponding to the set size for the trial. The outer positions were filled with “X”, which was never a memory item. Trials with different set sizes were presented in a random order, with the single exception that a trial with an incorrect response was always followed by a trial with a set size of four. After a maintenance period of three seconds, the probe letter was presented. The subjects responded with a button press to indicate whether or not the probe letter was contained in the stimulus (match IN/mismatch OUT). The subjects were instructed to respond as rapidly as possible without making errors. After the response, the probe was turned off, and the subjects received acoustic feedback regarding whether their response was correct or incorrect. The subjects performed 50 trials in one session, which lasted approximately 10 min. The task was performed using Presentation® software (Version 18.0, Neurobehavioral Systems, Inc., Berkeley, CA, www.neurobs.com). It is freely available at www.neurobs.com/ex_files/expt_view?id=266. During the recording period of several days, several subjects decided to perform more than one session of the task, up to seven sessions. The task ran on a notebook computer placed in front of the subject. Event transistor-transistor logic (TTL) pulses were sent to the ATLAS acquisition system via a parallel port.

### Subjects

Nine subjects participated in the study (Table 1). All subjects were patients with drug-resistant focal epilepsy and were implanted with depth electrodes in the medial temporal lobe for the potential surgical treatment of epilepsy. Implantation sites were selected solely based on the clinical indication. All subjects provided written informed consent for the study, which was approved by the institutional ethics review board (Kantonale Ethikkommission Zürich, PB-2016-02055). All subjects had normal or corrected-to-normal vision and were right-handed as confirmed by neuropsychological testing.

**Table 1:** Subject characteristics.

Subject number	Age	Sex	Handedness	Pathology	Implanted electrodes	Seizure onset zone (SOZ) electrodes	Number of sessions
1	24	F	Right	Xanthoastrocytoma WHO II	AHL,AL,ECL,LR,PHL,PHR	AHR,LR	4
2	39	M	Right	Gliosis	AHL,AHR,AL,AR,ECL,ECR,PHL,PHR	AHR,PHR	7
3	18	F	Right	Hippocampal sclerosis	AHL,AHR,AL,ECL,PHL	AHL,PHL	3
4	28	M	Right	Brain contusion	AHL,AHR,AL,AR,ECL,ECR,PHL,PHR	AHL,AHR,PHL,PHR	2
5	20	F	Right	Focal cortical dysplasia	AHL,AL,DRR,PHR	DRR	3
6	31	M	Right	Hippocampal sclerosis	AHL,AHR,AL,AR,ECL,ECR,PHL,PHR	AHL,PHL,ECL,AL	7
7	47	M	Right	Hippocampal sclerosis	AHL,AHR,AL,AR,ECL,ECR,PHL,PHR	AHR,PHR	4
8	56	F	Right	Hippocampal sclerosis	AHL,AHR,AL,AR,ECL,ECR,PHL,PHR	ECR	5
9	19	F	Right	Hippocampal sclerosis	AHL,AHR,AL,AR,ECL,ECR,PHL,PHR	ECR,AR	2

AH: hippocampal head; PH: hippocampal body; A: amygdala; EC: entorhinal cortex; L: left; R: right; LR: lesion; DRR: dysplasia.

### Recording setup

We measured iEEG with commercially available depth electrodes (1.3 mm diameter, 8 contacts of 1.6 mm length, spacing between contact centers 5 mm, ADTech®, Racine, WI, www.adtechmedical.com), implanted stereotactically into the hippocampus, entorhinal cortex and amygdala. Each macroelectrode had nine microelectrodes that protruded approximately 4 mm from its tip. Recordings were done against a common intracranial reference at a sampling frequency of 4 kHz for the macroelectrodes and 32 kHz for the microelectrodes via the ATLAS recording system (0.5–5000 Hz passband, Neuralynx®, Bozeman MT, USA, www.neuralynx.com). Scalp EEG electrodes were placed according to the 10–20 system with minor adaptations due to iEEG electrodes. Recordings were done at 256 Hz via the NicoletOne system (0.3–100 Hz passband, Natus®, https://neuro.natus.com). Scalp EEG and iEEG data were resampled at 200 Hz and 2 kHz, respectively.

### Depth electrode localization

Electrodes were localized using postimplantation CT scans and postimplantation structural T1-weighted MRI scans. Each scan was aligned to the ACPC (anterior commissure, posterior commissure) coordinate system. For each subject, the CT scan was registered to the postimplantation scan as implemented in FieldTrip^20,21^. In the coregistered CT-MR images, the electrode contacts were visually marked. The contact positions were normalized to the MNI space and assigned to a brain region using Brainnetome Atlas^22^. Due to subject-to-subject variability, sometimes an electrode seemed to be outside a structure in MTL. For these cases, we manually entered the anatomical structure for the electrode. In addition, depth electrode positions were verified by the neurosurgeon (L.S.) after merging preoperative MRI with postimplantation CT images of each individual subject in the plane along the electrode (iPlan Stereotaxy 3.0, Brainlab, München, Germany). We grouped electrodes according to whether they were recorded from the SOZ or another area. As an illustration, we projected the 3-dimensional positions of all electrode tips, i.e., the positions of the microelectrodes on 2-dimensional planes in the anatomical atlas (Fig. 2a–c).

### Spike detection and unit identification

The Combinato package (https://github.com/jniediek/combinato) was used for spike sorting^23^. Combinato follows a similar procedure to other freely available software packages: peak detection in the high-pass (>500 Hz) signal, computation of wavelet coefficients for detected peaks, and superparamagnetic clustering in the feature space of wavelet coefficients. As an advantage over other clustering procedures, Combinato provides better automated artifact rejection and is more sensitive in the detection of clusters of small size (few action potentials). We visually inspected each identified cluster based on the shape and amplitude of the action potentials and the interspike interval (ISI) distributions. We removed clusters that exhibited a low firing rate (<0.1 Hz), noisy waveforms, or nonuniform amplitude or shape of the action potentials in the recorded time interval. Moreover, to avoid overclustering, we merged highly similar clusters identified on the same microelectrode to obtain units. Finally, we computed several metrics of spike sorting quality (Fig. 2e–g). We refer here to a putative unit by the term ‘neuron’.

## Data Records

The dataset was released in the G-Node/NIX format and can be downloaded at 10.12751/g-node.d76994 ^24,25^. The README describes the repository structure and the instructions for downloading the data. Data from each session was saved in a single hierarchical data format (.h5) file. Standard NIX data structure was used, in addition to custom naming styles. Each file has the same format and includes: general information, information on the task, subject and session, scalp EEG data, intracranial EEG data, spike times and waveforms, and information on depth electrodes (Table 2).

**Table 2:** Data structure.

General information	Institution conducting the experiment
	Recording location
	Related publications (name, doi)
	Recording setup (devices and settings)
Task	Name
	Description
	URL for downloading the task for Presentation®
Subject	For each subject: Age, sex, handedness, pathology, depth electrodes, electrodes in seizure onset zone (SOZ)
Session	Session structure (number of trials per session, duration of trials)
	For each trial: trial number, set size, match/mismatch, correct/incorrect, response, response time, probe letter and trial rejection information
Scalp EEG data	For each trial: Signals recorded with a sampling frequency of 200 Hz
	Labels and time axis
Intracranial EEG data	For each trial: Signals recorded with a sampling frequency of 2 kHz
	Labels and time axis
Spike waveforms	For each unit: Mean and standard deviation of spike waveform in a 2-ms window, sampled at 32 kHz
Spike times	For each unit: Spike time with respect to t = 0 in the trial
Depth electrodes	MNI coordinates in millimeters
	Anatomical label
	Electrode label
	Anatomical label updated after visual inspection of MRI
	Electrodes in the seizure onset zone (SOZ)

## Technical Validation

### Behavior

The average correct response rates were 90.2% ± 7.8% for IN and 93.1% ± 4.3% for OUT. The rate of correct responses decreased with set size from a set size of 4 (98.5% correct responses) to set sizes of 6 (90.5%) and 8 (84.7%) (Fig. 1b, permuted repeated-measures ANOVA, F_2,70_ = 40.25, P = 0.0002). Across all sessions, the mean memory capacity was 6.8 (Cowan’s K^26^, (correct IN rate + correct OUT rate − 1)*set size), which indicates that the subjects were able to maintain at least 6 letters in memory. The mean response time (RT) for the correct trials (1630 trials) increased with workload (Fig. 1c, 48 ms/item, permuted repeated-measures ANOVA, F_2,2007_ = 21.10, P = 0.0002). Correct IN/OUT decisions were made more rapidly than incorrect decisions (1.36 ± 0.64 s versus 1.87 ± 1.21 s, respectively, permutation t-test, t = 5.06, P = 5.0 × 10^−5^). Together, the subjects performed the task within normal limits (7 ± 2 items) and easily performed the low-workload trials (set size 4)^2,27^. In sum, these data show that our subjects were able to perform the task and that the difficulty of the task increased with set size.

### Spike-sorting quality metrics

Spike sorting yielded single unit activity (SUA) and multiunit activity (MUA). We refer here to a putative unit by the term ‘neuron’. The example neuron in Fig. 2d increased its firing rate during maintenance. For all neurons, the histogram of the percentage of inter-spike intervals (ISI) < 3 ms is shown in Fig. 2e. The majority of neurons had less than 0.5% of short ISI. The percentage of ISI below 3 ms was 1.58 ± 4.18%. The histogram of average firing rate is given in Fig. 2f. For the mean waveform, the ratio of the peak amplitude to the standard deviation of the noise (waveform peak signal-to-noise ratio) was 3.28 ± 1.56 (Fig. 2g). These metrics are in the range of what is expected for the physiology of neuronal firing.

### Spectra of iEEG and scalp EEG signals

**Fig. 3:**
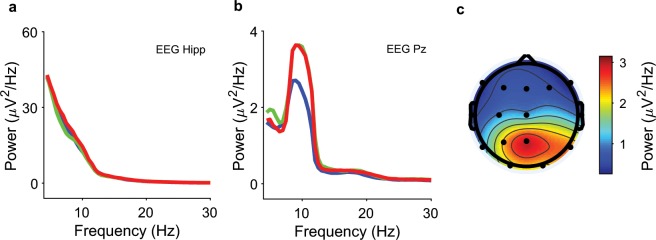
Spectra of EEG and iEEG during maintenance. Recordings from Subject 1 during the last 2 seconds of maintenance. **(a)** iEEG power from a left hippocampal contact (blue, set size 4; green, set size 6; red, set size 8); **(b)** scalp EEG power from electrode Pz; **(c)** scalp EEG power in the frequency band (8–12 Hz) for set size 8 highlights the parietal alpha rhythm.

The spectra for iEEG and scalp EEG power during the last two seconds of maintenance are given in Fig. 3a,b. Scalp EEG power in the alpha frequency band (8–12 Hz) for all contacts for set size 8 is given in Fig. 3c, showing parietal alpha power elicited during the maintenance period. The scalp EEG spectra agree with findings from healthy subjects with the same task^2^.

## Data Availability

An example script is provided with the dataset. It contains code snippets for reading and plotting the data and information related to the subjects and the task. All code is implemented in MATLAB (Mathworks Inc., version R2016B on Windows).
